# Dietetic Prescriptions in Bipolar Disorder: Nutritional Strategies to Support Mood Stability and Reduce Relapse Risk—A Narrative Review

**DOI:** 10.3390/life16010146

**Published:** 2026-01-16

**Authors:** Giuseppe Marano, Ester Maria Marzo, Greta Sfratta, Gianandrea Traversi, Esmeralda Capristo, Eleonora Gaetani, Marianna Mazza

**Affiliations:** 1 Department of Neuroscience, Head-Neck and Chest, Section of Psychiatry, Fondazione Policlinico Universitario Agostino Gemelli IRCCS, 00168 Rome, Italy; 2 Department of Neuroscience, Section of Psychiatry, Università Cattolica del Sacro Cuore, 00168 Rome, Italy; 3 Unit of Medical Genetics, Department of Laboratory Medicine, Ospedale Isola Tiberina-Gemelli Isola, 00186 Rome, Italy; 4 Department of Translational Medicine and Surgery, Fondazione Policlinico Universitario Agostino Gemelli IRCCS, Università Cattolica del Sacro Cuore, 00168 Rome, Italy; esmeralda.capristo@policlinicogemelli.it (E.C.);; 5 Department of Medical and Surgical Sciences, Fondazione Policlinico Universitario Agostino Gemelli IRCCS, 00168 Rome, Italy; 6 Unit of Internal Medicine, Cristo Re Hospital, 00167 Rome, Italy

**Keywords:** bipolar disorder, nutrition, dietary interventions, Mediterranean diet, omega-3 fatty acids, nutritional psychiatry, dietetic prescriptions

## Abstract

Background: Bipolar disorder (BD) is a chronic psychiatric condition characterized by recurrent mood episodes and substantial functional impairment. Emerging evidence highlights the role of nutrition in modulating neurobiological pathways and influencing the course of BD. However, systematic recommendations for dietetic prescriptions remain limited. Methods: This narrative review was conducted by searching PubMed, Scopus, and Web of Science up to October 2025. Keywords included “bipolar disorder,” “nutrition,” “dietary interventions,” and “nutritional psychiatry.” Studies focusing on nutritional patterns, dietary components, and dietetic recommendations relevant to BD were included. Evidence was synthesized narratively to identify potential dietary strategies and gaps in current knowledge. Results: The available literature suggests that nutritional interventions may influence mood stabilization, metabolic comorbidities, and treatment response in BD. Key findings highlight the potential benefits of Mediterranean-style diets, omega-3 fatty acids, micronutrients (including magnesium, zinc, and vitamins D and B-complex), and dietary approaches targeting inflammation and oxidative stress. Conversely, Western-style diets, rich in saturated fats and refined sugars, appear to exacerbate mood instability and metabolic burden. Despite promising findings, heterogeneity across studies and the scarcity of randomized controlled trials limit firm conclusions. Conclusions: Nutrition represents a promising adjunctive strategy in the management of BD. Dietetic prescriptions may contribute to improved outcomes by addressing both psychiatric symptoms and physical health comorbidities. Future research should prioritize well-designed clinical trials to establish evidence-based guidelines for integrating nutrition into BD management.

## 1. Introduction

### 1.1. Rationale for Dietary Approaches in Bipolar Disorder

Nutritional psychiatry is an emerging discipline that explores the relationship between dietary patterns and mental health disorders [1]. Although the impact of nutrition on physical health is well recognized, its role in mental health has historically received much less attention, despite mounting evidence that diet quality is a predictor of risk and outcomes in psychiatric conditions [2].

Diet and nutrition represent increasingly recognized modifiable factors in the prevention and management of mental disorders. Beyond caloric intake, qualitative aspects of diet, such as nutrient density, fatty acid composition, glycemic load, and exposure to pro- or anti-inflammatory food components, may influence neurobiological processes relevant to mood regulation.

Over the past decade, a growing body of observational and interventional research has shown that healthy dietary patterns, characterized by a high intake of vegetables, fruits, whole grains, nuts, seeds, and fish, are associated with a reduced risk of depression and improved symptom profiles, whereas diets rich in processed, high-fat, and high-sugar foods are linked to an increased vulnerability to depression and anxiety [2,3,4,5,6]. Meta-analytic findings confirm that dietary interventions can improve depressive symptoms across both clinical and non-clinical populations [7]. As poor diet and dysfunctional eating behaviors represent modifiable risk factors, dietary change has been increasingly recognized as a foundational treatment target, and recent clinical guidelines for mood disorders now include nutrition as a “non-negotiable” component of care [8,9].

Bipolar disorder (BD) is a severe and chronic mental illness characterized by recurrent mood episodes and substantial functional impairment. According to data from the Global Burden of Disease (GBD) studies, BD ranks among the leading causes of years lived with disability (YLDs) worldwide, particularly affecting individuals in early and middle adulthood, thereby exerting a disproportionate impact during the most productive years of life [9]. Recent GBD estimates indicate that BD contributes substantially to the global burden of mental disorders, with persistent disability despite treatment advances. Beyond its psychiatric manifestations, BD is associated with markedly increased all-cause mortality, largely driven by cardiovascular and metabolic comorbidities, as well as elevated suicide risk. The economic burden is equally significant, encompassing direct healthcare costs, reduced productivity, and indirect societal costs. These data underscore the urgent need for adjunctive, scalable, and preventive strategies capable of addressing both psychiatric symptoms and physical health outcomes in BD, including lifestyle-based interventions such as nutrition [9,10].

In BD, dietary patterns and nutrient intake have attracted growing interest due to their potential impact on inflammation, oxidative stress, mitochondrial function, and metabolic health, all of which are implicated in illness course and relapse risk. Framing nutrition as a multidimensional exposure encompassing both dietary patterns and individual nutrients is therefore essential to understanding its potential therapeutic role in BD.

In this disease, the rationale for dietary interventions is particularly compelling. Individuals with BD often exhibit unhealthy lifestyle habits, appetite fluctuations, and altered energy regulation, which may contribute to poor nutritional choices [10]. Furthermore, BD is associated with disproportionately high rates of general medical comorbidities, including diabetes, metabolic syndrome, cardiovascular disease, osteoporosis, and endocrine dysregulation, arising from both the intrinsic pathophysiology of the illness (e.g., systemic immuno-inflammatory activation, glucose–insulin abnormalities, oxidative stress, mitochondrial dysfunction) and the adverse effects of pharmacological treatments [11,12]. Dietary patterns and specific nutrients may influence these mechanisms: the Mediterranean diet exerts anti-inflammatory effects, omega-3 fatty acids modulate neuroplasticity, calorie restriction supports antioxidant defenses, while excessive sugar intake or gluten exposure can aggravate mitochondrial dysfunction and inflammatory processes [13].

So far, few studies have looked at the relationship between diet and BD, although it can be assumed that some of the observations about people with depression may be related to patients with BD. Observational data have nevertheless suggested that BD patients tend to consume more carbohydrates, with women also showing higher total energy intake [14] and that larger seafood consumption has been associated with a lower incidence of BD [15]. Additional studies have reported that people with BD may more frequently eat only one meal per day, face difficulties in sourcing and preparing food [16], and often display dietary patterns characterized by higher energy consumption and greater adherence to Western-type diets [17].

Overall, the interactions between diet and BD appear complex and multifactorial, potentially reflecting causal mechanisms, illness progression, lifestyle factors, and pharmacological influences. For example, the relationship between pharmacological treatment and obesity is well established [18], and unhealthy dietary choices may also represent a form of self-medication, as the preference for sugar-rich and fatty products could mitigate stress-induced cortisolemia in BD patients [13]. Importantly, these factors interact with the high burden of somatic comorbidities observed in BD, which are associated with therapeutic difficulties, drug resistance, and a more severe illness course [19]. In this context, appropriate dietary interventions may not only reduce the risks linked to diet-related medical conditions but also exert a beneficial effect on the trajectory of BD itself. Supporting this, patients with BD and type 2 diabetes or glucose intolerance show a greater risk of adverse illness course compared to those without glycemic abnormalities [20], and weight fluctuations have been linked to the occurrence of both manic and depressive episodes [19].

All these findings provide a strong biological and clinical rationale for considering nutrition as a relevant intervention target in BD.

### 1.2. Current Gaps in Treatment of Bipolar Disorder and Unmet Needs

Despite important advances in pharmacological and psychological therapies, BD continues to impose a substantial burden. Mental illness ranks among the leading causes of disability worldwide, accounting for 18.9% of years lived with disability [21], with an estimated $8.5 trillion in lost economic output attributed to mental, neurological, and substance use disorders [22]. Existing treatments, such as pharmacotherapy and psychotherapy, avert less than half of the disease burden [23,24], and despite broader access to psychotropics and psychological therapies, the population burden of depression, the predominant phase of BD, has not decreased and may even be rising [25]. In clinical practice, many patients with BD experience persistent symptoms, incomplete remission, and impaired functioning, leading to reduced quality of life [26].

Geographical variations in the incidence and prevalence of BD have prompted interest in the potential role of environmental and lifestyle factors, including diet. Ecological studies have reported lower rates of mood disorders in regions characterized by higher consumption of fish, fruits, vegetables, and unsaturated fats, such as Mediterranean and certain Asian countries, compared with regions where Western-style dietary patterns predominate. For instance, cross-national comparisons have suggested an inverse association between seafood consumption and BD prevalence, supporting a potential protective role of omega-3–rich diets. Conversely, regions with high intake of ultra-processed foods, refined carbohydrates, and saturated fats tend to exhibit higher rates of metabolic disorders and affective instability. While these associations cannot establish causality and are subject to confounding factors (e.g., genetic background, healthcare access, diagnostic practices), they provide a compelling epidemiological framework supporting further investigation of diet as a modifiable contributor to BD risk and course.

Patients consistently express a need for interventions that are effective, safe, and affordable [27]. However, research on the role of nutrition in BD remains limited, with early evidence largely derived from secondary analyses of studies not specifically designed to investigate nutritional outcomes [28]. Although recent findings underscore the relevance of diet in affective disorders, robust clinical data in BD are still scarce [29]. This gap highlights an urgent unmet need: integrating nutritional strategies into BD management has the potential to complement standard treatments, improve psychiatric outcomes, and simultaneously address the high burden of physical health comorbidities in this population. The objective of this narrative review is to synthesize current evidence on dietary patterns, specific nutrients, and bioactive compounds relevant to BD, with the aim of elucidating their potential mechanisms of action, clinical implications, and limitations, and to identify key gaps for future research.

## 2. Materials and Methods

This study was designed as a narrative review aimed at summarizing current evidence on dietetic prescriptions in BD. The review followed methodological recommendations for narrative syntheses in nutritional psychiatry.

### 2.1. Search Strategy

A systematic search of PubMed, Scopus, and Web of Science databases was conducted from inception to October 2025. The following keywords and Boolean operators were applied: “bipolar disorder” OR “manic-depressive illness” AND “nutrition” OR “diet” OR “dietary interventions” OR “nutritional psychiatry” OR “micronutrients” OR “dietetic prescriptions.” Additional references were identified through manual screening of bibliographies of relevant articles and reviews.

### 2.2. Eligibility Criteria

We included peer-reviewed studies addressing nutritional patterns, specific dietary components, or dietary prescriptions relevant to individuals with BD. Eligible study designs included clinical trials, cohort studies, case–control studies, cross-sectional analyses, systematic reviews, and meta-analyses. Exclusion criteria were: (1) studies not focused on BD, (2) preclinical/animal studies without translational relevance, (3) conference abstracts without full texts, and (4) articles not published in English.

Given the emerging nature of nutritional psychiatry in BD, this narrative review intentionally adopted broad inclusion criteria to capture early-phase evidence, including pilot studies and case reports, particularly for dietary approaches such as ketogenic diets where randomized controlled trials are scarce. Inclusion criteria comprised peer-reviewed human studies examining dietary patterns, nutritional interventions, or nutrient supplementation relevant to BD or, when explicitly stated, serious mental illnesses with mechanistic relevance. Exclusion criteria included non-English publications, conference abstracts without full texts, and purely preclinical studies lacking translational implications. A formal quantitative quality or risk-of-bias assessment was not performed, as this review was not designed as a systematic review. Instead, methodological limitations, sample size constraints, and potential biases of included studies are discussed narratively throughout the manuscript. This approach is consistent with narrative reviews aiming to map emerging evidence, generate hypotheses, and identify research gaps, while acknowledging the increased risk of selection bias inherent to non-systematic methodologies.

### 2.3. Data Extraction and Synthesis

Titles and abstracts were screened independently by two reviewers to assess relevance. Full-texts of eligible studies were examined, and data on dietary interventions, sample characteristics, outcomes, and main findings were extracted. Given the heterogeneity of study designs and outcomes, a narrative synthesis was performed. Results were organized thematically according to dietary patterns (e.g., Mediterranean diet, Western diet), specific nutrients (e.g., omega-3 fatty acids, vitamins, minerals), and dietetic recommendations relevant to clinical practice.

## 3. Pathophysiological Rationale

### 3.1. Inflammation, Oxidative Stress, and Metabolic Dysfunction

Chronic low-grade inflammation, characterized by elevated levels of pro-inflammatory cytokines and acute-phase proteins, has been consistently implicated in the development of depression, schizophrenia, and BD [12,26,27]. This inflammatory state is driven by multiple lifestyle factors, including psychological stress, smoking, obesity, poor sleep, and, critically, unhealthy diet [12]. Evidence from large observational studies indicates that adherence to healthy dietary patterns, such as the Mediterranean diet, rich in PUFA, fiber, fruits, and vegetables, is associated with lower concentrations of inflammatory markers [30]. Furthermore, intervention trials demonstrate that Mediterranean dietary patterns can significantly improve markers of inflammation. Meta-analyses and systematic reviews corroborate these findings, showing increased levels of interleukin-6 (IL-6), interleukin-8 (IL-8), tumor necrosis factor-alpha (TNF-α), and C-reactive protein (CRP) across major psychiatric disorders, including depression, anxiety, schizophrenia, and BD [31,32]. Elevated CRP levels and altered leukocyte profiles have also been observed in large cohort studies of depressive and anxiety disorders [33,34]. Importantly, anti-inflammatory dietary approaches, such as the Mediterranean diet, are associated with reductions in CRP, IL-6, and IL-1β, highlighting a plausible mechanistic link between diet, inflammation, and mental health outcomes [35,36].

Oxidative and nitrosative stress represent another critical pathway in the pathophysiology of mental illness [37]. Schizophrenia has been associated with decreased brain glutathione levels, altered glutamate metabolism, and heightened oxidative stress [38]. Similar abnormalities are reported in depression, where increased oxidative stress markers and reduced levels of key antioxidants, including vitamin E, vitamin C, coenzyme Q10, and glutathione, are found when compared with healthy controls [37]. Given the abundance of antioxidant compounds in fruits and vegetables, dietary strategies that enhance antioxidant availability may represent a feasible therapeutic approach.

Mitochondrial dysfunction is also closely linked to psychiatric disorders and may be particularly relevant to BD [39]. Impaired mitochondrial energy production, altered morphology, and disrupted distribution patterns have been described in depression and schizophrenia, potentially driven by a combination of reduced antioxidant capacity and cytokine-induced increases in mitochondrial-derived oxygen and nitrogen free radicals [40]. This interplay suggests that inflammation and oxidative stress may converge to drive mitochondrial impairment. Nutritional and nutraceutical compounds, including coenzyme Q10, α-lipoic acid, carnitine, creatine, resveratrol, and N-acetylcysteine (NAC), have been shown in animal models to up-regulate mitochondrial respiratory function [41,42]. Such findings highlight the relevance of dietary and nutraceutical interventions in targeting the interconnected mechanisms of inflammation, oxidative stress, and mitochondrial dysfunction that underlie psychiatric illness.

At the molecular level, oxidative stress and inflammation are regulated by redox-sensitive transcription factors, among which nuclear factor erythroid 2–related factor 2 (Nrf2) and nuclear factor kappa B (NF-κB) play a central role. Nrf2 is a master regulator of antioxidant defense, controlling the expression of genes involved in glutathione synthesis, detoxification enzymes, and mitochondrial protection. In contrast, NF-κB promotes the transcription of pro-inflammatory cytokines and mediators implicated in mood dysregulation. Dysregulation of the Nrf2–NF-κB balance has been described across serious mental illnesses, including BD, contributing to sustained neuroinflammation and oxidative damage. Several dietary components and micronutrients, such as omega-3 fatty acids, polyphenols, vitamins C and E, selenium, and magnesium, have been shown to activate Nrf2 signaling while inhibiting NF-κB pathways, thereby restoring redox homeostasis. These mechanisms provide a biologically plausible link between nutrition and mood stabilization in BD. In addition to peripheral inflammation, increasing evidence supports a role for neuroinflammation in BD. Activation of microglia and astrocytes, along with increased central cytokine signaling, has been reported across mood episodes and may contribute to synaptic dysfunction and mood instability. Peripheral inflammatory signals can cross or alter the blood–brain barrier, thereby amplifying neuroinflammatory processes. Dietary factors may influence neuroinflammation both directly, through bioactive compounds capable of crossing the blood–brain barrier, and indirectly, via modulation of systemic inflammation and gut–brain axis signaling.

**Figure 1:**
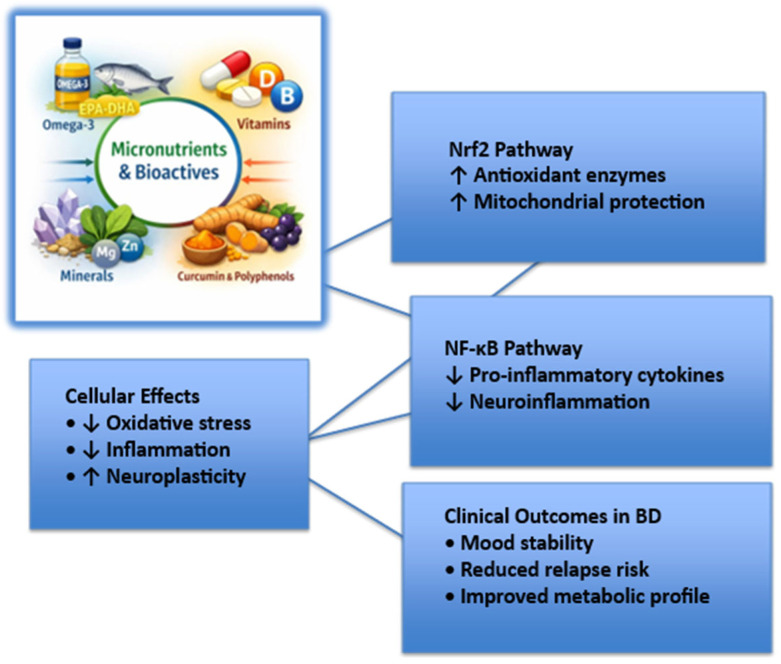
Molecular pathways linking micronutrients and bioactive compounds to mood stability in bipolar disorder.

To facilitate understanding of the molecular mechanisms through which nutritional factors may influence BD, Figure 1 provides a schematic overview of key redox-sensitive pathways, highlighting the balance between Nrf2-mediated antioxidant responses and NF-κB-driven inflammatory signaling, and their downstream cellular and clinical effects.

The figure illustrates how micronutrients and bioactive compounds may modulate redox balance and inflammatory signaling through activation of nuclear factor erythroid 2–related factor 2 (Nrf2) and inhibition of nuclear factor kappa B (NF-κB). These upstream molecular effects converge on cellular processes, including oxidative stress reduction, attenuation of neuroinflammation, and enhancement of neuroplasticity, ultimately contributing to improved mood stability and reduced relapse risk in bipolar disorder. Abbreviations: BD, bipolar disorder; Nrf2, nuclear factor erythroid 2–related factor 2; NF-κB, nuclear factor kappa B.

### 3.2. Gut–Brain Axis and Microbiome Considerations

The gut microbiota (GM) refers to the community of microorganisms that colonize the human body, while the term microbiome encompasses the genomes of all microorganisms inhabiting a given environment [43]. This complex ecosystem, shaped by intricate networks of positive and negative interactions, exerts a profound influence on host physiology and health [43]. Microorganisms are distributed throughout various body sites, including the skin, oral cavity, respiratory tract, and urogenital tract [44,45], though the majority, up to 90%, reside in the gastrointestinal (GI) tract, particularly in the small and large intestines [46,47]. The GM is composed not only of bacteria but also fungi, viruses, protists, and archaebacteria, the latter thriving in extreme anaerobic environments through metabolic processes such as methanogenesis [48,49].

Diet is among the most important determinants of microbiota composition and diversity [50]. Nutrients such as vitamins, minerals, polyunsaturated fatty acids (PUFAs), and amino acids play essential roles in metabolic pathways, neurotransmitter synthesis, and immune regulation, thereby influencing both gut and brain function. Microbial fermentation of dietary fiber produces short-chain fatty acids (SCFAs), key metabolites implicated in gut–brain communication and neuroinflammation [51,52]. Clinical evidence supports diet–microbiome–mood interactions [53,54,55]. It has been reported that adherence to a Mediterranean diet supplemented with fish oil reduced depressive symptoms compared to control conditions [56]. On the other hand, the effects of vegetarian and vegan diets on mental health remain inconsistent, with some studies suggesting increased risk of depression, others reporting benefits, and some showing no association at all [57].

Mechanistic insights from animal and human studies highlight multiple pathways through which the GM may influence mental illness. The microbiota modulates neurobiological systems relevant to psychiatric disorders, including brain-derived neurotrophic factor (BDNF) signaling [58,59], serotonergic neurotransmission [60], immune function [61], and hypothalamic–pituitary–adrenal (HPA) axis regulation [58,62]. Germ-free mice display exaggerated stress responses and reduced cortical and hippocampal BDNF and serotonin receptor expression compared to conventionally colonized mice [58,63], whereas stress itself alters microbial composition in animal models [64]. Translational studies support a bidirectional relationship: patients with depression exhibit reduced microbial richness and diversity [65], while transplantation of fecal microbiota from depressed individuals induces depression-like behavior in rodents [64,66]. Moreover, modulation of the microbiome through probiotics or diet has been shown to alter depression-related behaviors in preclinical models [67].

Diet-induced changes in intestinal permeability also represent a potential pathway linking GM to mood disorders. High-fat diets compromise the integrity of tight junctions in the intestinal epithelium [68], increasing translocation of bacterial products such as lipopolysaccharides into circulation. This, in turn, triggers immune activation, cytokine release, and nitro-oxidative stress pathways, thereby amplifying systemic inflammation and potentially contributing to psychiatric symptoms [69,70]. The gut–brain axis provides a biologically plausible framework for understanding how nutrition influences psychiatric outcomes, including those relevant to BD. By modulating microbial composition and function, diet emerges as a critical determinant of neuroimmune and neuroendocrine processes implicated in mood regulation. Fermented dairy products, such as yogurt and kefir, have attracted interest due to their probiotic content and potential effects on the gut–brain axis. In observational and interventional studies of depression and other serious mental illnesses, consumption of fermented dairy products has been associated with improved metabolic parameters and, in some cases, reduced depressive symptoms. These effects are thought to be mediated by modulation of gut microbiota composition, enhancement of intestinal barrier integrity, and reduction of low-grade inflammation. However, data specific to BD remain limited, and existing evidence should be interpreted cautiously. Further bipolar-specific studies are needed to determine whether fermented dairy products confer mood-stabilizing or relapse-preventive benefits.

## 4. Dietary Patterns and Nutritional Interventions

Dietary patterns represent a key modifiable lifestyle factor that may influence the course, prognosis, and clinical outcomes of BD. Conversely, unhealthy dietary patterns may exert adverse effects on mood regulation and illness course. Diets characterized by a high intake of ultra-processed foods, refined sugars, saturated fats, and low fiber content, commonly referred to as Western-style diets, have been consistently associated with increased systemic inflammation, oxidative stress, insulin resistance, and gut microbiota dysbiosis. These biological alterations may exacerbate mood instability, contribute to the persistence of depressive symptoms, and increase cardiometabolic burden, which in turn negatively affects prognosis, treatment response, and overall functional outcomes in BD. In contrast, dietary patterns rich in whole foods, unsaturated fats, fiber, and bioactive compounds have been proposed as potentially beneficial adjunctive strategies, targeting both the neurobiological mechanisms of BD and its frequent metabolic comorbidities.

### 4.1. Mediterranean Diet and Anti-Inflammatory Approaches

Growing evidence suggests that dietary patterns may influence the course and symptomatology of BD. The Mediterranean diet, first described by Ancel Keys, is characterized by a high intake of fruits, vegetables, unrefined cereals, olive oil, nuts, and seafood; moderate consumption of poultry, dairy products, and red wine; and a low consumption of red meat [71,72]. Beyond its recognized cardiometabolic benefits, adherence to this dietary pattern has been associated with a reduced risk of several Axis I psychiatric disorders [73,74,75].

Several mechanisms may underlie these effects. The Mediterranean diet is rich in antioxidants, omega-3 fatty acids, B vitamins, magnesium, and polyphenols, which contribute to lowering oxidative stress and systemic inflammation, both implicated in the pathogenesis of BD [76,77,78,79,80].

Increased intake of fruits and vegetables within this dietary pattern has been associated with decreased levels of inflammatory markers such as interleukin-6 and C-reactive protein [81,82,83]. Moreover, adherence to the Mediterranean diet has been linked to higher serum levels of BDNF, supporting neurogenesis and synaptic plasticity [84,85]. By contrast, Western-type diets, characterized by high consumption of processed food, sugars, and saturated fats, have been associated with reduced hippocampal volume and impaired neurogenesis [86,87,88]. These findings suggest that the Mediterranean diet may exert a protective role in BD through anti-inflammatory and antioxidant effects, modulation of neurotrophic factors, and preservation of neurogenesis [89,90]. While further clinical studies specifically targeting BD populations are needed, current evidence highlights this dietary pattern as a promising adjunctive strategy in the management of the disorder.

### 4.2. Ketogenic and Low-Carbohydrate Diets

The defining characteristic of a ketogenic diet (KD) is the production of ketones, including acetoacetic acid, β-hydroxybutyric acid, and acetone, which serve as an alternative energy substrate for the central nervous system and peripheral tissues [91,92]. The KD is characterized by high fat (65–80% of energy), moderate protein (20–25%), and very low carbohydrate content (5–10%), mimicking the metabolic effects of fasting [91,92]. Initially developed in the 1920s for the treatment of drug-resistant epilepsy [93], the KD has since evolved to address a variety of medical conditions, including neurological and psychiatric disorders [94,95,96].

In BD, evidence remains preliminary but promising. A pilot interventional study demonstrated that euthymic BD patients could successfully adhere to a modified KD for 6–8 weeks, achieving and maintaining ketosis with high compliance and only minor side effects [97]. Case reports have described sustained mood stabilization in individuals with bipolar II disorder who maintained ketosis for two to three years [98], with improvements surpassing those achieved with pharmacotherapy, and even remission in a patient who was able to discontinue quetiapine and reduce lamotrigine dosage following KD adoption [99]. Proposed mechanisms include effects on ionic channel regulation, increased blood acidity, modulation of GABAergic signaling, and inhibition of glutamatergic AMPA receptors by medium-chain fatty acids. However, not all reports are consistent: in one case study, lack of ketosis was associated with no observable benefit [100].

Beyond case-level data, small clinical and observational studies further support potential benefits. In a retrospective study of 12 patients with bipolar II disorder, a strict KD (<20 g carbohydrates/day) during psychiatric hospitalization was associated with improvements in weight, blood pressure, triglycerides, LDL cholesterol, as well as reductions in depressive and psychotic symptoms. An online observational study of 274 individuals with BD found that KD users reported greater and more sustained mood stabilization compared to other dietary interventions, alongside improvements in energy, cognition, and weight regulation [101]. More recently, a four-month clinical survey in BD patients with metabolic abnormalities reported significant improvements in global clinical impression, with 69% of participants showing >1 point improvement and the majority achieving recovery or remission when adherence was high [102].

Mechanistically, ketone bodies may bypass mitochondrial dysfunction implicated in BD pathophysiology, improving neuronal bioenergetics. This is consistent with evidence of altered energy metabolism and a “Warburg effect” profile in BD, including changes in isocitrate dehydrogenase, α-ketoglutarate dehydrogenase, pyruvate, and lactate, as well as reduced N-acetylaspartate levels in drug-naïve patients [103,104]. These metabolic shifts suggest that KD may target core pathophysiological processes in BD rather than acting solely on symptoms. Taken together, emerging but limited evidence supports the feasibility and potential efficacy of KD as an adjunctive intervention in BD. While preliminary clinical studies and case reports suggest benefits in mood stabilization, metabolic health, and functional outcomes, larger randomized controlled trials are required to establish its therapeutic role.

### 4.3. Plant-Based Diets

Dietary patterns are often categorized as omnivore or plant-based. While omnivore diets typically include high amounts of arachidonic acid, an animal-derived fatty acid associated with lower mood [105,106], plant-based diets emphasize fruits, vegetables, whole grains, soy, nuts, and seeds, with vegan diets excluding all animal products and vegetarian diets permitting dairy or eggs. Importantly, both omnivorous and plant-based diets can vary substantially in quality: low-quality versions, characterized by high sugar, saturated fats, and refined grains, may adversely affect mental health [107].

The relationship between plant-based diets and depressive symptoms remains equivocal. Some studies report higher depression scores in vegetarians and vegans compared to omnivores [108,109], (while others suggest the opposite, with lower depressive symptoms among plant-based groups [105,110,111]. Systematic reviews confirm these inconsistencies [112,113], raising the possibility that diet quality, rather than dietary pattern itself, is the more relevant determinant of mental health [37,114]. In this regard, a recent Australian study by Walsh et al. (2023) [115] found that a high-quality plant-based diet was associated with fewer depressive symptoms, whereas a low-quality plant-based diet was linked to more severe symptoms [111]. These results align with broader evidence that high-quality diets protect against depression [2].

So far, most research has focused on unipolar depression, and the relevance of these findings to BD remains uncertain. Given that depressive phases are central to the course and burden of BD, and that diet quality influences inflammatory and oxidative stress pathways implicated in its pathophysiology, future studies should assess whether the benefits of high-quality plant-based diets extend to patients with BD. Conversely, potential risks associated with nutritional deficiencies (e.g., vitamin B12, omega-3 fatty acids) in poorly balanced plant-based diets warrant careful consideration, especially in populations vulnerable to mood instability.

**Figure 2:**
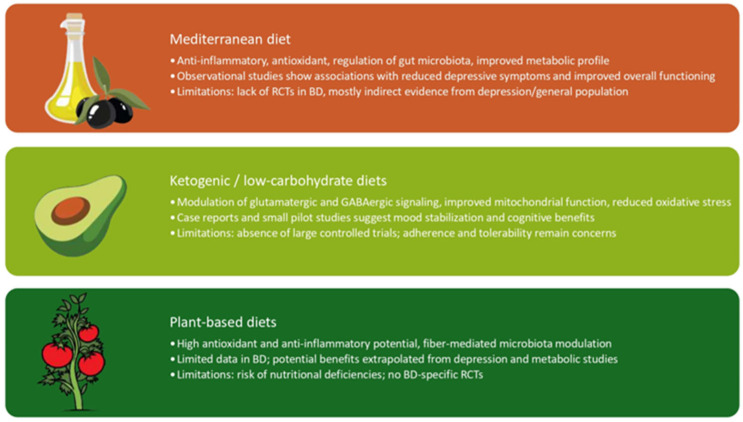
Summary of major dietary approaches explored in Bipolar Disorder.

Figure 2 provides a schematic overview of the Mediterranean, ketogenic/low-carbohydrate, and plant-based diets, summarizing their main biological mechanisms, supporting evidence, and current limitations.

## 5. Specific Nutrients and Supplementation

Beyond global dietary patterns, research has also focused on the contribution of individual nutrients and their potential use as adjunctive supplementation strategies in BD. The rationale stems from evidence that specific micronutrients and bioactive compounds influence biological processes central to BD pathophysiology, including neuroinflammation, oxidative stress, mitochondrial function, and neurotransmitter regulation [11,12]. Oral supplementation is of particular interest because it represents a relatively safe, accessible, and low-cost intervention that may target residual symptoms, enhance treatment response, and mitigate comorbid medical risks [20,27]. Among the most extensively studied are omega-3 fatty acids, vitamins such as folate and vitamin D, essential minerals including magnesium and zinc, and selected amino acids and nutraceuticals, each with varying degrees of supporting evidence in BD.

### 5.1. Omega-3 Fatty Acids

Epidemiological and biochemical studies have consistently shown that individuals with BD present reduced consumption and lower blood and brain levels of omega-3 fatty acids, particularly EPA and DHA, suggesting a potential role in illness pathophysiology [116]. A higher dietary omega-6/omega-3 ratio, frequently observed in BD, has been linked to elevated peripheral inflammation [117,118] while higher levels of linoleic acid (LA) and other PUFAs appear to correlate with improved outcomes, including reduced depressive symptoms and fewer suicide attempts [60,119]. Omega-3 fatty acids are crucial for cell membrane structure, neurotransmission, and anti-inflammatory processes, and experimental evidence indicates that they may modulate serotonin and dopamine signaling, increase GABA activity, and inhibit neuronal pathways similarly to established mood stabilizers such as lithium and valproate [120,121,122,123].

Despite these mechanistic premises, clinical findings remain mixed. Interventional studies indicate only modest or inconsistent effects of omega-3 supplementation in BD. Some small trials suggested benefits in depressive or manic symptomatology [62,67], whereas others did not find significant improvements on standard rating scales or relapse prevention [63,66,109,124]. A narrative review by Psara et al. (2025) [125] concluded that while evidence points toward a potentially favorable role for omega-3 supplementation in BD, current clinical studies are limited by small sample sizes, short follow-up, and heterogeneity in design and dosing regimens. The authors emphasize that omega-3s may hold greater value as a prophylactic strategy rather than in acute episodes, but robust evidence is still lacking. Therefore, larger and methodologically rigorous trials are warranted to establish efficacy, clarify interactions with standard pharmacological treatments, and evaluate long-term outcomes. Synthetic omega-3 analogs are also under investigation as possible adjunctive treatments [125,126]. Emerging evidence suggests that EPA and DHA may exert partially distinct effects. EPA appears to have stronger anti-inflammatory properties and has been more consistently associated with improvements in depressive symptoms, whereas DHA plays a predominant role in neuronal membrane structure, synaptic function, and neurodevelopment [125]. In mood disorders, including bipolar disorder, EPA-dominant formulations have generally shown greater efficacy in reducing depressive symptomatology, while DHA may contribute more to long-term neurobiological resilience. Nonetheless, direct comparative trials in BD are scarce, and optimal EPA:DHA ratios remain to be established.

### 5.2. Vitamins (e.g., Folate, Vitamin D, B-Complex)

Vitamin D, traditionally known for its role in bone ossification, has been increasingly recognized as a neurosteroid with implications for mood regulation [127,128]. Its receptors are expressed in the brain, where vitamin D contributes to neurogenesis, neurotransmission, synaptic plasticity, HPA axis modulation, and immune regulation [129,130,131,132,133,134,135]. Observational studies have linked low serum 25(OH)D levels to increased risk of depression [136], including perinatal depression [137]. Meta-analytic evidence remains mixed: Mikola et al. (2023) [138] concluded that supplementation exerts a small but significant effect on depressive symptoms, particularly in Major Depressive Disorder (MDD) and perinatal depression, with limited benefits in healthy populations. These results are consistent with prior findings of stronger efficacy in MDD [139,140], especially at higher doses and in deficient individuals [136,141].

With regard to BD, the literature remains inconclusive. Cereda et al. (2021) [142] found no clear association between vitamin D and BD, although hospitalized patients consistently displayed lower serum concentrations [143,144,145,146] suggesting a link with acute illness phases. Phase-specific analyses indicate that vitamin D deficiency may correlate with greater depressive severity [145,147] and that supplementation could reduce symptoms in both depressive and manic episodes, albeit in small samples [143,147,148]. These findings support the hypothesis that vitamin D may modulate neuroinflammation and immune dysregulation observed during BD exacerbations [149,150,151,152]. Nevertheless, causality remains uncertain, as low vitamin D levels could either reflect or contribute to the pro-inflammatory state typical of acute episodes [153].

Beyond vitamin D, evidence also highlights the role of folate (B9) and vitamin B12. Faugere et al. (2025) [154] demonstrated that deficiencies in these vitamins are associated with impaired neurotransmitter synthesis, elevated homocysteine, oxidative stress, and metabolic dysfunction in patients with schizophrenia, MDD, and BD. In BD specifically, Lam et al. (2022) [155] reported that folate supplementation, either as folic acid or bioactive forms such as L-methylfolate, can enhance treatment response in both manic and depressive phases, though gene–nutrient interactions (e.g., MTHFR polymorphisms) and drug–nutrient interactions (e.g., with lamotrigine) may moderate efficacy. Safety data suggest that active folate derivatives are well tolerated and may represent a promising adjunctive strategy in BD management.

Current evidence indicates that vitamin deficiencies, particularly vitamin D, folate, and B12, are prevalent in BD and may influence clinical severity, treatment response, and metabolic comorbidities. While observational studies strongly suggest an association, interventional trials remain limited and heterogeneous. Longitudinal and precision-based studies are needed to clarify causal relationships and optimize supplementation strategies tailored to individual biological and genetic profiles.

### 5.3. Minerals (e.g., Magnesium, Zinc, Selenium)

Several minerals have been investigated in relation to psychiatric disorders, although their role in BD remains uncertain. Magnesium (Mg^2+^), which is involved in multiple neurobiological processes including neurotransmission and synaptic plasticity [156,157], has shown antidepressant-like effects in preclinical models via modulation of NMDA receptors, the HPA axis, and monoaminergic systems [158,159]. Clinical findings in depression and other psychiatric conditions suggest a possible synergistic effect with antidepressants [160,161], but randomized trials are scarce and overall inconclusive. Importantly, no consistent evidence supports the use of magnesium supplementation in BD [162]. Zinc, another essential micronutrient regulating enzymatic activity, immune response, and glutamatergic neurotransmission [163,164,165,166], has been more extensively studied in mood disorders. While decreased serum zinc is consistently observed in major depression [167], findings in BD are inconsistent. Some studies report lower zinc levels during depressive episodes [168,169,170], whereas higher levels have been described in mania [145]. A large study of clinically stable BD patients reported elevated zinc concentrations compared to controls, without associations with clinical severity or inflammatory markers [171]. There is evidence for disturbed zinc homeostasis in BD, but the direction of change appears mood-state dependent, and supplementation studies are lacking. In addition, despite these biochemical alterations, clinical studies on mineral supplementation in BD are absent, and no evidence currently supports the therapeutic use of oral mineral supplementation in this population. Table 1 provides an overview of key nutrients, their proposed mechanisms, evidence base, and relevance for clinical practice.

### 5.4. Curcumin

Curcumin, a polyphenolic compound derived from *Curcuma longa*, has attracted increasing interest for its neuroprotective and psychotropic properties. Preclinical and clinical studies suggest that curcumin exerts antioxidant, anti-inflammatory, and neurotrophic effects through modulation of Nrf2 activation, inhibition of NF-κB signaling, reduction of pro-inflammatory cytokines, and upregulation of BDNF. In depressive disorders, curcumin supplementation has been associated with improvements in mood symptoms, particularly as an adjunct to standard antidepressant treatment [3]. Although data specific to BD remain limited, the shared pathophysiological mechanisms across serious mental illnesses, particularly oxidative stress and inflammation, support the potential relevance of curcumin as an adjunctive nutraceutical. Issues related to bioavailability, dosing, and interaction with pharmacological treatments warrant careful consideration in BD populations.

When discussing interventions with specific nutrients, it is important to note that reported effects generally refer to doses administered as supplements in clinical or experimental studies, rather than to amounts achievable through diet alone. Supplement doses varied widely across studies and were often higher than typical dietary intake. Given the heterogeneity of dosing regimens, study designs, and patient populations, this review does not propose specific supplementation doses. Instead, nutrient amounts are discussed descriptively to contextualize existing evidence, underscoring the need for well-designed dose-finding trials in BD.

## 6. Integration into Clinical Practice

### 6.1. Dietary Counseling and Collaboration with Dietitians

Individuals with BD present a markedly increased risk of obesity, metabolic syndrome, and cardiovascular diseases compared to the general population [172,173]. These risks are further exacerbated by poorer general health, higher prevalence of comorbid conditions, and the metabolic side effects of many pharmacological treatments commonly prescribed in BD [172,173,174]. Lifestyle interventions that include dietary counseling have shown effectiveness in reducing BMI, body weight, triglycerides, and fasting glucose [175,176], while also contributing to improvements in psychosocial well-being, quality of life, and symptom severity [177,178]. Within clinical practice, collaboration between psychiatrists and dietitians is critical to address nutritional needs in a structured way, providing individualized dietary plans, practical guidance on food selection, and strategies for sustainable weight management. Such interdisciplinary care can mitigate the cardiometabolic burden of BD and should be considered an integral part of treatment pathways, particularly given that structured dietary interventions have demonstrated greater benefits than treatment as usual or brief psychoeducation [179,180].

### 6.2. Patient Adherence and Lifestyle Barriers

Despite encouraging evidence, a major obstacle to the effectiveness of dietary interventions is patient adherence. Residual depressive symptoms, fatigue, low motivation, and social isolation often undermine patients’ capacity to engage with and sustain lifestyle changes [181]. Data from lifestyle programs for individuals with serious mental illness indicate high attrition rates, with nearly half of participants not attending all sessions in the first six months, and adherence dropping even further over longer follow-up periods. Depressive states in particular have been linked to lower adherence to lifestyle modifications after medical events such as myocardial infarction [182]. High dropout rates have also been reported in randomized controlled trials of lifestyle interventions in BD, representing a significant limitation to generalizability [183,184]. To address these barriers, evidence suggests that motivational interviewing, structured goal-setting, and individualized problem-solving strategies are crucial for sustaining engagement [185]. Digital tools, such as smartphone applications and wearable devices, may further support adherence by enabling real-time monitoring of dietary and physical activity behaviors, although their role in BD requires further study [186]. The therapeutic alliance between clinicians and patients has also emerged as a key factor, as participants often report that the relationship with the therapist strengthens their motivation to adhere to lifestyle changes.

### 6.3. Personalized and Precision Nutrition Approaches

The impact of dietary interventions in BD is not uniform across patient groups. Evidence from the LIFESTYLE trial [187] related studies indicates that sociodemographic and clinical characteristics, including gender, educational attainment, and baseline psychosocial functioning, significantly shape outcomes [188,189]. Women and more educated patients tend to report greater improvements in body mass index (BMI), weight, and waist circumference, whereas individuals with poorer psychosocial functioning benefit less, possibly due to lower participation in social and group activities. These findings underline the importance of personalized interventions that adapt both content and delivery to patients’ characteristics and needs. Precision nutrition approaches represent a promising avenue for future care, incorporating metabolic profiles, comorbidities, and pharmacological regimens to optimize dietary recommendations. Moreover, motivational strategies and multicomponent designs that address not only diet but also physical activity, circadian rhythms, smoking habits, and adherence to medication have been associated with greater and more persistent improvements [185,190]. Tailoring interventions to individual risk profiles and motivational levels could therefore enhance both efficacy and adherence. In clinical practice, this translates into the need for stratified treatment models, where dietary counseling and lifestyle modification plans are delivered in a way that reflects the biological, psychological, and social complexity of BD.

Based on the current body of evidence, some preliminary clinical recommendations can be made, although substantial research gaps remain. Table 2 summarizes practical recommendations and highlights areas where further investigation is required.

### 6.4. Nutritional Assessment Tools in Serious Mental Illnesses

Accurate assessment of dietary intake and nutritional status represents a critical step in implementing nutritional interventions in serious mental illnesses (SMIs), including BD. Traditional clinical evaluation often underestimates dietary inadequacies in psychiatric populations, where cognitive symptoms, motivational deficits, and socioeconomic barriers may complicate self-reporting. In recent years, validated nutritional assessment tools have increasingly been adapted or developed for use in SMIs. Food frequency questionnaires (FFQs), 24 h dietary recalls, and diet quality indices are now widely used to characterize habitual dietary patterns in patients with mood and psychotic disorders [191]. Importantly, SMI-specific adaptations of FFQs have been proposed to account for irregular eating behaviors, meal skipping, and high consumption of ultra-processed foods commonly observed in these populations. In addition to self-report measures, objective assessments such as anthropometric parameters, bioimpedance analysis, and laboratory evaluation of micronutrient status (e.g., vitamin D, B-complex vitamins, iron, zinc, and omega-3 fatty acids) provide complementary information. Integrating structured nutritional assessments into routine psychiatric care may facilitate early identification of deficiencies, guide personalized dietary prescriptions and improve monitoring of treatment response.

### 6.5. Food–Medication and Micronutrient–Medication Interactions

Food–medication and micronutrient–medication interactions represent a clinically relevant but often overlooked aspect of nutritional interventions in BD. Mood stabilizers and antipsychotics may influence appetite, weight, glucose metabolism, and micronutrient status, while dietary components can, in turn, modulate drug absorption, metabolism, and efficacy. These interactions underscore the importance of integrating nutritional assessment into routine pharmacological management and of coordinating dietary interventions with prescribing clinicians to optimize both efficacy and safety [1,2].

Drug–nutrient interactions are particularly relevant in BD, where long-term pharmacotherapy is the cornerstone of treatment. Lithium pharmacokinetics are strongly influenced by sodium and fluid intake, such that abrupt dietary sodium restriction or dehydration may increase the risk of lithium toxicity. Valproate is associated with weight gain, insulin resistance, and alterations in lipid metabolism, potentially amplifying the metabolic impact of unhealthy dietary patterns, while nutritional strategies emphasizing glycemic control and anti-inflammatory components may mitigate these effects [120]. Lamotrigine metabolism may be indirectly influenced by folate status, and interactions with high-dose folate supplementation have been suggested, although clinical implications remain uncertain [155]. These considerations underscore the importance of coordinating dietary interventions with pharmacological management to optimize safety and treatment outcomes.

### 6.6. Digital Health Technologies

Digital health technologies are emerging as promising tools to enhance the effectiveness of nutritional interventions in serious mental illnesses. Artificial-intelligence-based analytics, combined with data derived from wearable devices and smartphone applications, allow continuous monitoring of dietary intake, physical activity, sleep–wake cycles, and circadian rhythm stability, factors closely linked to mood regulation in BD. Machine-learning algorithms can integrate multimodal data streams to identify early warning signals of mood destabilization and relapse risk, potentially enabling timely dietary and lifestyle adjustments. Smart nutrition apps can support patients through personalized meal planning, real-time feedback, and adherence tracking, while also reducing clinician burden. Although evidence specific to BD remains limited, early studies suggest that digital phenotyping and AI-driven approaches may improve engagement, self-management, and preventive care [13].

The integration of dietary recommendations into routine psychiatric care requires consideration of adherence challenges and psychosocial barriers common in BD. Residual depressive symptoms, cognitive impairment, low motivation, social isolation, and socioeconomic constraints may limit patients’ capacity to adopt and sustain dietary changes. Moreover, mood fluctuations can disrupt routines and eating behaviors, reducing long-term adherence. Multidisciplinary approaches involving dietitians, psychoeducation, and motivational strategies are therefore essential. Gradual, flexible dietary modifications and realistic goal-setting may enhance feasibility, particularly during euthymic phases. Digital tools and structured lifestyle programs may further support adherence, although their effectiveness in BD requires further evaluation.

## 7. Conclusions and Future Directions

As highlighted throughout this review, emerging evidence on dietary interventions in BD is highly promising but remains partial. Observational and interventional studies suggest beneficial effects of overall dietary patterns, such as the Mediterranean diet, ketogenic diets, and well-balanced plant-based diets, on mood regulation, neuroinflammation, oxidative stress, and metabolic health. Conversely, Western-type diets, rich in processed foods, sugars, and saturated fats, have been associated with increased vulnerability to mood disturbances and impaired neurogenesis. Evidence on micronutrient and macronutrient supplementation, including omega-3 fatty acids, vitamins D and B-complex, and minerals such as magnesium and zinc, also indicates potential benefits for modulating neurotransmitter pathways, neuroinflammation, and oxidative stress, but remains inconclusive due to small sample sizes, heterogeneous study designs, and short follow-up periods. Integration of dietary strategies into clinical practice requires structured dietary counseling, interdisciplinary collaboration, and strategies to enhance adherence, considering lifestyle barriers, psychosocial functioning, and individual risk profiles. Personalized or precision nutrition approaches, tailored to metabolic profiles, comorbidities, and pharmacological regimens, appear essential to maximize clinical benefits.

A limitation of the current literature is that much of the evidence informing nutritional strategies in BD is extrapolated from studies conducted in other serious mental illnesses, particularly major depressive disorder and schizophrenia. While these conditions share overlapping pathophysiological mechanisms, such as inflammation, oxidative stress, metabolic dysregulation, and circadian rhythm disturbances, they also differ substantially in clinical course, treatment response, and neurobiological substrates. Consequently, findings derived from unipolar depression or schizophrenia cannot be directly generalized to BD without caution. In this review, such evidence is discussed within the broader framework of serious mental illnesses, acknowledging both shared mechanisms and disorder-specific gaps. This highlights the urgent need for bipolar-specific nutritional research to validate and refine current hypotheses.

In addition, it is pivotal to outline that the potential effects of dietary interventions in BD may vary according to illness phase. Depressive phases, which account for the greatest proportion of illness burden, appear most amenable to nutritional strategies targeting inflammation, oxidative stress, and metabolic dysfunction, such as Mediterranean-style diets or omega-3 supplementation. During manic or hypomanic phases, evidence is more limited, and dietary interventions should primarily aim at metabolic stabilization and harm reduction rather than acute symptom control. In euthymic phases, nutritional strategies may play a preventive role by supporting mood stability, reducing relapse risk, and mitigating long-term cardiometabolic comorbidities. Overall, current evidence suggests that dietary interventions are most consistently relevant during depressive and euthymic phases, while their role in acute mania remains largely unexplored.

It is important to distinguish between associative findings derived from observational studies and evidence from interventional trials. Much of the literature on dietary patterns and micronutrients in BD is observational in nature and therefore cannot establish causality. In contrast, interventional evidence, particularly randomized controlled trials, remains limited and heterogeneous, especially for micronutrient supplementation and plant-based dietary patterns. While observational studies provide valuable hypotheses and biological plausibility, clinical recommendations should primarily be guided by interventional data, which are currently strongest for certain dietary patterns and weaker for isolated nutrient supplementation.

Future research should aim to expand study populations, adopt rigorous, long-term, and controlled designs, and clarify the optimal dietary composition, nutrient dosing, and timing. While definitive conclusions are still lacking, the current findings provide a strong rationale for continued investigation. Nutritional interventions have the potential to complement standard pharmacological and psychological treatments, mitigate somatic comorbidities, and ultimately improve both psychiatric and physical health outcomes in patients with BD.

## Figures and Tables

**Table 1:** Nutrients and dietary components relevant to Bipolar Disorder.

Nutrient/Component	Proposed Mechanism	Evidence Type	Relevance to BD	References
Omega-3 fatty acids (EPA, DHA)	Anti-inflammatory, membrane stabilization	Multiple RCTs (depression, some in BD)	Adjunctive benefit, esp. for depression	Sarris et al. 2012 [1]
Folate/B vitamins	Homocysteine regulation, neurotransmitter synthesis	Observational, small trials	Mixed findings, possible stabilization	Lam et al. 2022 [155]
Vitamin D	Neuroimmunomodulation, neurotrophins	Observational	Deficiency common; unclear supplementation benefit	Lin et al. 2020 [37]
Magnesium	NMDA receptor modulation, neuroprotection	Case reports, small trials	Possible mood stabilization	Botturi et al. 2020 [162]
Zinc, selenium	Antioxidant, enzymatic regulation	Limited data	Deficiency linked to mood symptoms	Siwek et al. 2016 [170]

Abbreviations: EPA = Eicosapentaenoic acid; DHA = Docosahexaenoic acid; NMDA = N-methyl-D-aspartate; BD = Bipolar disorder; RCT = Randomized controlled trial.

**Table 2:** Clinical recommendations and gaps in evidence.

Recommendation	Evidence Support	Clinical Applicability	Research Gaps
Encourage Mediterranean-style diet	Strong indirect evidence	Safe, broadly applicable	BD-specific RCTs needed
Consider ketogenic diet in refractory cases	Weak (case reports, pilots)	Limited by adherence	Larger controlled studies required
Ensure adequate omega-3 intake	Moderate evidence	Safe, feasible	More BD-focused RCTs needed
Monitor deficiencies in restrictive diets	Clinical consensus	Important in long-term care	Prospective BD-specific trials

Abbreviations: BD = Bipolar disorder; RCT = Randomized controlled trial.

## Data Availability

No new data were created or analyzed in this study. Data sharing is not applicable to this article.
